# A non-randomized pilot study to test the feasibility of developing a frailty scale for pet cats

**DOI:** 10.3389/fvets.2025.1549566

**Published:** 2025-02-26

**Authors:** Elizabeth J. Colleran, Mikel M. Delgado, Yunyi Ren, Alexander J. German, Margaret E. Gruen, Danièlle A. Gunn-Moore, Kathleen Romanowski, Wendy Simpson, Christine Kirnos, Kathleen Keefe Ternes, Judy Karnia, Marybeth Temples, Sandra L. Taylor, Melissa Bain, C. A. Tony Buffington

**Affiliations:** ^1^Chico Hospital for Cats, Chico, CA, United States; ^2^Feline Minds Cat Behavior Consulting, Sacramento, CA, United States; ^3^Department of Public Health Sciences, School of Medicine, University of California, Davis, Davis, CA, United States; ^4^Institute of Life Course and Medical Sciences, Faculty of Health and Life Sciences, University of Liverpool, Neston, United Kingdom; ^5^Department of Clinical Sciences, College of Veterinary Medicine, North Carolina State University, Raleigh, NC, United States; ^6^The Royal (Dick) School of Veterinary Studies and The Roslin Institute, The University of Edinburgh, Easter Bush Veterinary Campus, Edinburgh, United Kingdom; ^7^Department of Surgery, School of Medicine, University of California, Davis, Sacramento, CA, United States; ^8^Morrisville Cat Hospital, Morrisville, NC, United States; ^9^The Cat Hospital of Media, Media, PA, United States; ^10^The Feline Hospital, Salem, MA, United States; ^11^Scottsdale Cat Clinic, Scottsdale, AZ, United States; ^12^Civic Feline Clinic, Walnut Creek, CA, United States; ^13^Department of Medicine and Epidemiology, School of Veterinary Medicine, University of California, Davis, Davis, CA, United States

**Keywords:** feline, senior, comorbidities, quality of life, end of life, palliative care

## Abstract

**Introduction:**

Human frailty has long been studied and dozens of “frailty scales” have been developed, but equivalent research is more limited in cats. This pilot study aimed to determine the feasibility of recruiting and retaining veterinary practices and owners, collecting study data, and analyzing results about frailty in older cats.

**Methods:**

Participating feline-exclusive practice veterinarians recruited cats aged 11–20 years, of either sex and of any breed. Owners completed a questionnaire about their cat and estimated its frailty. Study veterinarians also estimated the cat's frailty after obtaining a history, conducting a physical examination, and completing a separate questionnaire. The derived variables were used to investigate the following domains of frailty: (1) cognitive function; (2) behavior; (3) activity; (4) body weight; (5) body condition score; (6) muscle condition score; (7) any unexplained changes in weight, cognitive function, or eating behavior; and (8) the number of chronic diseases identified in the cat. Some cats were followed prospectively for 6 months, and mortality during this period was compared with frailty status, as determined by the veterinarian.

**Results:**

Half (6/12) of the veterinary practices invited to participate successfully recruited 273 owner-cat pairs, with baseline questionnaire results obtained from 189 owners (69%) and veterinarian questionnaires obtained for 210 cats (77%). Of 122 cats having both owner and veterinarian questionnaire results, 45 (37%) were classified as frail by the owner and 51 (42%) by the veterinarian, with 28 (23%) classified as frail on both questionnaires. Of the cats with follow-up data, 13 of the 64 cats (20%) reported by veterinarians to be frail died or were euthanased during the 6-month follow-up, compared with only 1 of 54 cats (2%) that were not reported to be frail (Fisher's exact test *P* = 0.003).

**Discussion:**

Developing a brief feline frailty questionnaire (FFQ) was feasible, and the results of such assessments were associated with 6-month mortality. A larger definitive trial should be considered to explore further the (dis)agreement between owners and veterinarians and better understand which frailty signs owners might be missing.

## 1 Introduction

Frailty describes variable combinations of reduced functional reserve capacity, decreased physiological and cognitive performance, and increased vulnerability to adverse health outcomes that can occur with advancing age (1). The concept gained attention in human medicine and health policy because of its prevalence and predictive capacity for adverse outcomes such as falls, fractures, dependency on others to perform daily activities, hospitalizations, and death (1).

Human frailty has been studied for many years, and dozens of “frailty scales” have been developed. The Frailty Phenotype (1) and the Frailty Index (2) are two of the most used clinical frailty assessments. The Frailty Phenotype (1) measures five specific criteria: unintentional weight loss, weakness, poor endurance, slowness, and low activity, whereas the Frailty Index (2) measures deficits in physiological, psychological, cognitive, and social function. These assessments identify 5–10% of community-dwelling individuals aged 65 years or older as frail, with the prevalence of frailty increasing with age (3). Both prefrailty (score 1–2/5) and frailty (score 3–5/5) predicted a decline in activities of daily living compared with robustness (score 0/5) in people (4). Fortunately, frailty is not a unidirectional process; in humans, frailty improves with appropriate interventions (5, 6).

To understand the nature and underlying biological mechanisms of frailty, objective measures have been designed for preclinical models that are similar to human frailty assessments (7). The mouse clinical frailty index is based on the cumulative deficit model, with quantification of 31 deficits (8). The mouse frailty phenotype tool, eqivalent to the frailty phenotype developed in humans (1), uses performance measures to define frailty as the presence of three or more criteria, including weight change, endurance, weakness, slow walking speed, and low physical activity. Several versions of the mouse frailty phenotype assessment have been developed. One frailty phenotype was validated in aged C57BL/6 mice and included grip strength, walking speed, physical activity, and endurance that were evaluated by the inverted-cling grip test, rotarod, voluntary wheel running, and an endurance score obtained from the grip test and rotarod (9). Weight change was not considered in this model. This frailty phenotype was refined by Kane et al. (10), to include consideration of weight loss, and by Bauman et al. (11) to consider weight gain. In this respect.

Baumann et al. (11), measured body weight, walking speed, strength, endurance, and physical activity in 28 male mice every 5 months starting at 14 months of age. At 23 months of age, nine of the 28 mice were either pre-frail (*n* = 6) or frail (*n* = 3). In contrast, Kwak et al. (10), studied 27 female mice using a similar protocol. They found that five mice were pre-frail and five were frail at 20 months of age. These studies used the Frailty Phenotype approach to identify frailty, measuring voluntary wheel running, a treadmill test, a grip meter, and walking speed using equipment unavailable in primary care clinical veterinary medicine.

Frailty also has been observed in pet cats. For example, Gunn-Moore proposed a Mobility/Cognitive Dysfunction Questionnaire for geriatric cats in 2011 (12), whilst Bellows et al. described frailty in geriatric cats as “multisystem impairment associated with increased vulnerability to stressors and increased risk of adverse health outcomes” (13). Several related “quality of life” scales have been proposed for pet cats (14–19), including for cats under hospice care (20), but, to the authors' knowledge, the concept of frailty in older cats has not yet been developed further.

The concept of frailty has also been examined in dogs (21–25), including one “frailty index” (24), a quantitative score that increases with the degree of frailty, and two “frailty phenotype assessment tools” (23), which utilize different criteria (e.g., weakness, exhaustion, low physical activity, chronic undernutrition, and poor mobility), based on owner assessment, veterinarian assessment or both to assign different phenotypes (e.g., “frail” and “robust”) (21, 22, 25). However, to date, frailty has not been widely studied in cats.

People of similar ages often have very different functional abilities (26), and the authors speculate that cats also vary in functional abilities as they age. Thus, whereas age alone is not a good predictor of outcomes for individual cats, it is likely that the prevalence of frailty also increases with age in cats. Although “senior” is defined as over 10 years of age in the 2021 American Animal Hospital Association (AAHA) and American Association of Feline Practitioners (AAFP) Feline Life Stage Guidelines (27), the 2021 AAFP feline senior care stage guidelines (28) document states that “some cats may be more appropriately referred to as senior as early as 8 years of age, possibly sooner for some breeds or those with genetic predispositions.” However, since many cats are adopted or rescued, their true ages are often unknown. Therefore, age alone cannot adequately measure a cat's health, physiological or behavioral functioning, or needs.

Dent et al. (29) suggested that frailty measurements should (1) accurately identify frail subjects, (2) be supported by a biological causative theory, (3) be simple to use, (4) reliably predict adverse clinical outcomes, and (5) reliably predict patient responses to stressors and therapies. This study aimed to investigate the feasibility of developing a feline frailty questionnaire (FFQ) for use by owners and veterinarians in feline practice and undertake some preliminary validation of such a system.

Pilot studies are essential for planning larger definitive trials because they can be used to investigate proposed methods and to determine whether more extensive trials are feasible (14). Recognizing the necessity for further frailty research in cats, this study aimed to determine the feasibility of such studies, including recruiting and retaining veterinary practice and owner participants, collecting study data, using matched data from multiple sources to increase reliability and reduce bias, and analyzing results. It also provided an opportunity to analyze potential differences between owner and veterinarian assessments of cat frailty to identify possible areas for owner education (15).

## 2 Materials and methods

### 2.1 Study timeframe and ethical considerations

The study commenced in March 2020, was paused during the COVID-19 pandemic, and recommenced in September 2021, before completion in March, 2023. The study used non-experimental (i.e., client-owned) cats who were given established, internationally-recognized high standards (“best practice”) of veterinary clinical care. As a result, it was deemed that no IACUC approval was required. Nevertheless, informed, written consent was obtained from all owners. Given that only anonymized data were analyzed and reported, additional informed consent for publication was not required.

### 2.2 Cats and eligibility

One of the authors (EJC) invited 12 feline-exclusive veterinary practices based in the USA to participate, six of which successfully recruited owner-cat pairs for the study. The practices were in various locations across the USA, including: Morrisville, NC; Media, PA; Salem, MA; Scottsdale, AZ; and Chico and Walnut Creek, CA. The main eligibility criterion was that the cat should be client-owned and between 11 and 20 years old; to be as representative as possible of the population under study, there were no other specific inclusion or exclusion criteria.

### 2.3 Study design

The study followed a recognized two-phase procedure for scale development (16). Questions were pre-tested to determine the extent to which (1) they reflected the domain being studied and (2) their answers were valid measurements of the domain. Cats were recruited given their likelihood of presenting with (pre)-frailty based on advanced reported age.

The owner questionnaire was pre-tested by owners of 30 cats in the target age group. They were asked to complete the questionnaire and provide feedback about which questions were confusing, difficult to understand, or otherwise difficult to answer. This feedback was used to modify the questionnaire for clarity, and the revised version (Supplementary File 1) had the following reading statistics: Flesch reading ease 76.4 (fairly easy to read), Flesch-Kincaid reading grade level 4 (average reading level), and passive sentences 3.4% (good readability). During the pilot study, owners completed this questionnaire before the veterinarian examined their cat. In contrast, veterinarians only estimated the cat's frailty after obtaining a history, performing a physical examination, and completing a veterinarian questionnaire (Supplementary File 2).

### 2.4 Measures

A list of variables created by consensus of four study authors (AJG, MG, DGM, KR) with experience in feline aging and frailty, in conjunction with a review of published questionnaires (5, 6, 17–19), was used to generate candidate FFQ items for the owner and veterinarian questionnaires. Content validity was assessed by ensuring that all items had a generally accepted definition, the domains were clearly defined, each domain was relevant to the frailty construct, experts agreed that the domains were adequately sampled and that the domain dimensions could be reliably observed and evaluated.

### 2.5 Statistical analysis

All cats in the study underwent re-evaluations 6 months apart and were followed from enrollment until the cat died, was lost to follow-up, or was reported still to be alive at the conclusion of the study. Cats with both owner and veterinarian questionnaire data were randomly assigned to training and validation sets in a 70:30 ratio. To enhance the owner data in the training set, we included additional data from 66 cats for which only owner questionnaires were available. Similarly, the veterinary data in the training set was augmented by adding data from 85 cats where only veterinarian questionnaires had been completed. As a result, the training set for the owner questionnaire comprised 152 cats, whilst that for the veterinary questionnaire comprised 171 cats. Using these augmented training sets, univariate analyses of cat frailty for the owner and veterinarian questionnaires were first conducted separately and summarized before differences between cats identified as frail or not (by the questionnaire respondent, owner or veterinarian) were tested. For numeric outcomes, two-sample *t*-tests were used, whilst Fisher's exact test and Chi-squared tests were used as appropriate for categorical variables. The *P-*values derived from these univariate analyses were corrected for false discovery rate (FDR) using the Benjamini-Hochberg procedure. Given the concern that variables with significant *P*-values, but not significant FDR values, could be false positives, the FDR values were considered in selecting variables to include in the modeling (see below).

Outcome variables were not tested for normality because most were binary variables, where a normality assumption is not applicable. The only two continuous outcomes (in univariable analysis) were owner-reported age and years owned, and both were analyzed with *t*-tests, which are robust to departures from normality, for moderate to large sample sizes, provided that there are no large outliers and the data are generally symmetrical on visual inspection. Since both owner-reported age and years owned met these conditions, we decided that the use of a formal normality test was not necessary.

The following variables were used to develop a multivariable prediction model of frailty using owner survey responses (Supplementary File 3): owner-reported cat age (< 16 years, >16 years); hesitates or avoids climbing or jumping up onto or down from objects; grooms less than usual; changes in eating habits over the past 3 months; responds slower; behavior composite score1 and behavior composite score 2. These variables were selected based on consideration of statistical significance and those considered most reliably reportable by owners. Behavior composite 1 was the sum of the following 16 behaviors that owners noticed changes in, adding one to the composite score for each behavior that was marked as “Yes”: plays with toys either less or no longer; moves without purpose; stares into space; gets lost at home; avoids interactions; clings to you; cries out loudly; acts more fearful; acts agitated or restless; cries when picked up; poops outside the litterbox; pees outside the litterbox; plays less; sleeps more; explores less; active at night. Behavior composite score 2 was created by removing four behaviors (“avoids interactions”, “clings to you”, “acts agitated or restless”, and “active at night”), included in Behavior composite score 1, from the composite score. This was because these behaviors were, instead, included individually in the model.

For modeling, items from the veterinarian questionnaire included owner-reported cat age (< 16 years, >16 years), muscle condition score (MCS), claw condition, increase in fatigue or cognitive difficulties in the past 3 months, and an additional disease variable, which was either chronic kidney disease (CKD; yes, no) or a disease composite score. The disease composite score was constructed from the responses to disease binary (yes, no) indicators (cancer; neurological disease; chronic pain; cognitive dysfunction syndrome [CDS]; dental disease; dermatological disease; gastrointestinal disease; cardiovascular disease; hyperthyroidism; hypothyroidism; CKD; chronic lower urinary tract disease; endocrine disease; and hypertension), by adding one to the composite score for every disease that was marked as “Yes.” Multivariable logistic regression models were fitted to both the owner and veterinarian training sets to develop initial predictive models for frailty. To address possible multicollinearity issues in the owner questionnaire data, either behavior composite score 1 or 2 (see above) was included in the variable set, meaning that the two variable sets were assessed separately. Similarly, to address possible multicollinearity in the veterinarian questionnaire data, either CKD or the disease composite score was included in the variable set; therefore, again, two different variable sets were evaluated.

For each variable set, all variables were initially included, and the model was then refined using a stepwise selection procedure based on the Bayesian Information Criterion (BIC) for selecting variables, in conjunction with the *P*-value, whereby *P* < 0.15 was applied as a cut-off for exclusion. Both *P*-values and BIC were used for variable selection because this enhances robustness of the model fitting process: the *P*-values are used to identify which variables enter the model in the first place, as well as which remain in the model during the backward step; the BIC is then used to evaluate overall fit (generalizability) of the model considering all variables. A key advantage of BIC, over other information criteria such as the Akaike Information Criterion, is that it penalizes model complexity more; thus, BIC helps to identify parsimonious models (30). Two different variable sets were evaluated for each questionnaire (Supplementary File 3). The stepwise selected models were then refitted using Firth's bias reduction method to address the complete separation observed for some variables. Model performance was evaluated by calculating the area under the receiver operating characteristic (AUROC) curve derived using the validation set; sensitivity, specificity, positive (PPV), and negative (NPV) predictive values for all probability thresholds were also determined.

Finally, to provide some preliminary validation of the FFQ, Fisher's exact test was used to compare mortality in cats initially classified as either frail or not frail based on veterinarian classification that were followed for 6 months. Unfortunately, it was not possible to conduct a more detailed survival analysis, for example, using Cox's proportional hazards regression or plotting Kaplan-Meier curves, because specific dates of death or censoring for each cat were not recorded and, therefore, exact survival times could not be calculated.

Statistical analyses were performed using open-source statistical software (R, version 4.2.2; Foundation for Statistical Computing, Vienna, Austria) (31), with additional packages “logistf version 1.26.0” (32) and “pROC” version 1.8.5 (33), for Firth's logistic regression modeling and AUC calculation, respectively. The level of statistical significance was set at *P* < 0.05, and all analyses were two-sided. The datasets and statistical code used for statistical analyses are included as Supplementary Files 4, 5.

## 3 Results

### 3.1 Study population and questionnaire data

A total of 273 client-owned cats were recruited, with baseline questionnaire results obtained within 6 months from 189 (69%) of these owners. Veterinarian questionnaires were obtained for 210 cats (77%) performed within 30 days of the cat's examination. Altogether, 122 cats had both owner and veterinarian baseline questionnaire records. Of these cats, 62 were female (62 neutered), and 60 were male (59 neutered); their mean owner-reported age was 14 y (standard deviation, SD 2.1 y). Most (111) were non-pedigree, with the remaining 11 cats being purebred. Of the 122 cats where both questionnaires were completed, 45 (37%) and 51 (42%) were classified as frail by owner and veterinarian, respectively, with 28 (23%) cats being classified as frail by both owner and veterinarian.

### 3.2 Owner questionnaire

Of the 152 cats in the owner questionnaire training set, 50 (33%) were classified as frail. In univariate statistical analyses, 14 variables differed significantly by frailty status (frail or not, Table 1); all such variables differed significantly based on both *P-*values and FDR values.

**Table 1:** Comparison of results from cats classified as frail or not frail in the owner questionnaire.

**Variable**	**Group** ^**a**^		**Total (152)**	***P*-value^b^**	**FDR^c^**
	**Not Frail (102)**	**Frail (50)**			
**1. Age in years (rounded to nearest year)** ^d^				< 0.001	< 0.001
Mean (SD)	13 (1.9)	15 (2.1)	14 (2.1)		
Range	10–17	11–20	10–20		
**2. Jump up onto elevated surfaces, like chairs or tables?**				< 0.001	< 0.001
Unchanged	76 (74.5%)	19 (38.0%)	95 (62.5%)		
Diminished/ceased	26 (25.5%)	31 (62.0%)	57 (37.5%)		
**3. Climb, such as onto furniture or a cat tree?**				< 0.001	< 0.001
Unchanged	80 (80.0%)	15 (33.3%)	95 (65.5%)		
Diminished/ceased	20 (20.0%)	30 (66.7%)	50 (34.5%)		
Missing	2	5	7		
**4. Use a scratching post?**				< 0.001	0.003
Unchanged	77 (81.9%)	19 (48.7%)	96 (72.2%)		
Diminished/ceased	17 (18.1%)	20 (51.3%)	37 (27.8%)		
Missing	8	11	19		
**5. Have a good appetite?**				< 0.001	< 0.001
Unchanged	80 (78.4%)	21 (42.9%)	101 (66.9%)		
Diminished/ceased	22 (21.6%)	28 (57.1%)	50 (33.1%)		
Missing	0	1	1		
**6. Sleep or rest comfortably?**				< 0.001	0.019
Unchanged	101 (99.0%)	42 (84.0%)	143 (94.1%)		
Diminished/ceased	1 (1.0%)	8 (16.0%)	9 (5.9%)		
**7. Look out the window?**				< 0.001	0.016
Unchanged	79 (83.2%)	25 (55.6%)	104 (74.3%)		
Diminished/ceased	16 (16.8%)	20 (44.4%)	36 (25.7%)		
Missing	7	5	12		
**8. Enjoy life?**				< 0.001	< 0.001
Unchanged	91 (89.2%)	25 (52.1%)	116 (77.3%)		
Diminished/ceased	11 (10.8%)	23 (47.9%)	34 (22.7%)		
Missing	0	2	2		
**9. Respond slower than usual when called?**				< 0.001	< 0.001
No	94 (92.2%)	28 (56.0%)	122 (80.3%)		
Yes	8 (7.8%)	22 (44.0%)	30 (19.7%)		
**10. Hesitate/avoid jumping up onto or down from objects (for example, onto your lap, chair, or couch)?**				< 0.001	< 0.001
No	75 (73.5%)	19 (38.0%)	94 (61.8%)		
Yes	27 (26.5%)	31 (62.0%)	58 (38.2%)		
**11. Move less smoothly than usual?**				< 0.001	< 0.001
No	78 (76.5%)	16 (32.0%)	94 (61.8%)		
Yes	24 (23.5%)	34 (68.0%)	58 (38.2%)		
**12. Groom her/himself less than usual?**				< 0.001	0.003
No	87 (85.3%)	28 (56.0%)	115 (75.7%)		
Yes	15 (14.7%)	22 (44.0%)	37 (24.3%)		
No	82 (80.4%)	21 (42.0%)	103 (67.8%)		
Yes	20 (19.6%)	29 (58.0%)	49 (32.2%)		
**14. Changes in weight in the previous 3 months? (no, gain, loss)**				< 0.001	0.018
No	61 (59.8%)	15 (30.0%)	76 (50.0%)		
Yes	41 (40.2%)	35 (70.0%)	76 (50.0%)		

^a^Cats were classified as “frail” or “not-frail” according to the opinion of the owner. ^b^Reported P-values for comparisons according to frailty status, tested either by Chi-square or Fisher's exact tests, for categorical variables, or two-sample t-tests for continuous numeric variables. ^c^Original P-values corrected for false-discovery rate (FDR) using the Benjamini-Hochberg procedure. SD = standard deviation. ^d^Cat age was reported by the owner and was not verified.

Using stepwise multiple logistic regression, the odds of an owner classifying their cat as frail were positively associated with three variables in the final best-fit model: “hesitates or avoids climbing or jumping,” “change in eating habits” and “responds slower” (Table 2). Frail cats were older than not frail cats, whilst owners reported greater proportions of frail cats with diminished or ceased activities for: “jumps onto elevated surfaces”, “climbs”, “uses scratching post”, “has good appetite”, “sleeps or rests comfortably”, “looks out window” and “enjoys life”. Further, more frail cats were reported to “respond slower than usual when called”, “hesitate/avoid jumping up or down”, “move less smoothly than usual”, “groom less than usual”, “have changes in eating in the last 3 months” and “have changes in weight in the last 3 months”.

**Table 2:** Results of multiple logistic regression analyses for the variables retained in the final owner model.

**Variable**	**Estimate**	**Odds ratio**	**95% confidence intervals**		**Pr (>|z|)**
			**Lower**	**Upper**	
(Intercept)	−2.791				
Hesitates or avoids climbing or jumping	1.733	5.66	2.33	13.71	< 0.001
Change eating habits	1.766	5.85	2.39	14.30	< 0.001
Responds slower	2.119	8.32	2.80	24.72	< 0.001

Results are reported as estimates of regression coefficients (β), with odds ratios, 95% confidence intervals, and the respective P-value from a Z-test [Pr(>|z|)]. Variables listed are those retained after stepwise selection.

When applied to the validation set, the owner model had modest discriminatory ability (AUROC 0.763, 95% confidence interval [95%-CI] 0.600, 0.927; Table 3). Sensitivity, specificity, PPV, and NPV over the range of predicted probabilities for frailty from the final owner model are shown in Table 4.

**Table 3:** Sensitivity, specificity, positive predictive value (PPV), and negative predictive value (NPV) for the final owner model.

**Frailty threshold**	**Sensitivity**	**Specificity**	**PPV**	**NPV**
0.000	1.000	0.000	0.322	-
0.158	1.000	0.495	0.485	1.000
0.261	0.771	0.752	0.597	0.874
0.301	0.625	0.871	0.698	0.830
0.504	0.562	0.901	0.730	0.812
0.706	0.375	0.960	0.818	0.764
0.746	0.250	0.980	0.857	0.733
0.847	0.229	0.990	0.917	0.730
1.000	0.000	1.000	-	0.678

Results are for multiple regression analyses using the final owner model, where different frailty thresholds derived from the owner training set were applied to the owner validation set.

**Table 4:** Area under the receiver operating characteristic curve for the training and validation sets for the final models.

**Model name**	**Training set**	**Validation set**
Owner model set 1^a^	0.861 [0.805, 0.917]	0.763 [0.600, 0.927]
Veterinarian model set 1^b^	0.778 [0.715, 0.840]	0.789 [0.675, 0.904]
Veterinarian model set 2^c^	0.853 [0.793, 0.914]	0.819 [0.675, 0.963]

Results are area under the receiver operating characteristic curve for both the training and validation sets.

^a^The variables retained in evaluating owner model sets 1 and 2 were the same; ^b^included chronic kidney disease as a predictor; ^c^included disease composite score as a predictor.

### 3.3 Veterinarian questionnaire

Veterinarian questionnaire data were available for 171 cats; 69 (40%) were classified as frail. In univariate analyses, seven variables differed significantly by frailty status after adjusting for multiple tests (Table 5). Given possible multicollinearity, CKD and disease composite scores were tested separately along with the other variables (variable set 1: with CKD; variable set 2: with disease composite score).

**Table 5:** Comparison of results from cats classified as frail or not frail in the veterinarian questionnaire.

**Variable**	**Group** ^**a**^		**Total (152)**	***P*-value^b^**	**FDR^c^**
	**Not Frail (102)**	**Frail (50)**			
**Muscle condition score**				< 0.001	< 0.001
Normal, Mild Loss	100 (98.0%)	37 (53.6%)	137 (80.1%)		
Moderate, Severe Loss	2 (2.0%)	32 (46.4%)	34 (19.9%)		
**Claw condition**				< 0.001	< 0.001
Normal	94 (92.2%)	37 (53.6%)	131 (76.6%)		
Ingrown, overgrown, or declawed	8 (7.8%)	32 (46.4%)	40 (23.4%)		
**Cognitive dysfunction syndrome (CDS)**				< 0.001	0.008
No	102 (100.0%)	61 (88.4%)	163 (95.3%)		
Yes	0 (0.0%)	8 (11.6%)	8 (4.7%)		
**Chronic kidney disease (CKD)**				< 0.001	< 0.001
No	79 (77.5%)	24 (34.8%)	103 (60.2%)		
Yes	23 (22.5%)	45 (65.2%)	68 (39.8%)		
**In the last 3 months, has this cat: (Yes** = **yes or don't know, No** = **no)**					
**Involuntarily lost weight?**				< 0.001	< 0.001
No	75 (78.1%)	23 (37.1%)	98 (62.0%)		
Yes	21 (21.9%)	39 (62.9%)	60 (38.0%)		
Missing	6	7	13		
**Been more fatigued?**				< 0.001	< 0.001
No	96 (100.0%)	44 (67.7%)	140 (87.0%)		
Yes	0 (0.0%)	21 (32.3%)	21 (13.0%)		
Missing	6	4	10		
**Shown increased cognitive difficulties?**				< 0.001	0.007
No	96 (100.0%)	55 (87.3%)	151 (95.0%)		
Yes	0 (0.0%)	8 (12.7%)	8 (5.0%)		
Missing	6	6	12		
**Shown signs of cognitive dysfunction?**				< 0.001	< 0.001
No	96 (100.0%)	51 (81.0%)	147 (92.5%)		
Yes	0 (0.0%)	12 (19.0%)	12 (7.5%)		
Missing	6	6	12		

^a^Cats were classified as “frail” or “not-frail” according to the opinion of the owner. ^b^Reported P-values for comparisons according to frailty status, tested either by Chi-square or Fisher's exact tests. ^c^Original P-values corrected for false-discovery rate (FDR) using the Benjamini-Hochberg procedure.

Using stepwise multiple logistic regression, muscle loss and fatigue were retained from both variable sets; however, while disease composite score was also retained in variable set 2, CKD was not retained from variable set 1. This was because the disease composite used in variable set 2 explained more variance in the frailty outcome than CKD by itself. Overall, in the best-fit model, the odds of a veterinarian classifying a cat as frail were positively associated with three variables: muscle loss, fatigue, and disease composite (Table 6). Significantly more frail cats were reported to have a MCS of moderate or severe loss; ingrown, overgrown or declawed claw condition; cognitive dysfunction syndrome, and CKD. Further, veterinarians reported more frail cats to have had involuntarily lost weight, been more fatigued, to have shown increased cognitive difficulties and shown signs of cognitive dysfunction in the last 3 months.

**Table 6:** Results of the final multiple logistic regression analysis for the variables from the veterinarian questionnaire.

**Variable**	**Estimate**	**Odds ratio**	**95% confidence intervals**		**Pr (>|z|)**
			**Lower**	**Upper**	
(Intercept)	−2.425				
Muscle loss	3.059	21.30	5.16	87.88	< 0.001
Fatigue	3.514	33.58	1.92	586.85	< 0.001
Disease composite score	0.459	1.58	1.17	2.14	0.002

Results are reported as estimates of regression coefficients (β), with odds ratios, 95% confidence intervals and the respective P-value from a Z-test [Pr(>|z|)]. Variables listed are those retained after stepwise selection.

This final veterinarian model (AUROC 0.819, 95% confidence interval 0.675, 0.963) was marginally better than the performance of both the final owner model and the model using variable set 1 (Table 4). Sensitivity, specificity, PPV, and NPV over the range of predicted probabilities for frailty from the best-fit veterinarian model are shown in Supplementary File 6.

### 3.4 Association between 6-month mortality and frailty in cats

Six-month follow-up data were available from 118 cats, 64 of which (54%) were classified by the veterinarian as frail, with the remaining 54 cats (46%) classified as not frail. Of the 64 cats defined as frail, 13 (20%) died within 6 months, whilst only one (2%) of the 54 cats defined as not frail died within 6 months (Fisher's exact test *P* = 0.003).

## 4 Discussion

The results of this pilot study demonstrated that developing a feline frailty questionnaire was feasible and provided valuable information to inform the development of a larger definitive study. In this respect, owners were successfully recruited and retained in the study, with baseline questionnaire results being obtained within 6 months from 94% of the recruited owners, with 84% of those owners submitting at least one additional questionnaire. Half of the feline-exclusive veterinary practices, from across the United States, recruited cases for the study, and veterinarian questionnaires were obtained for 77% of cats within 30 days of their examination. Future studies would benefit from a dedicated study support technician to ensure more timely questionnaire submissions and to facilitate better ongoing communications with practice leadership and staff regarding study objectives and progress (26). Better compensation for practices' time, resources, and space may also improve practice engagement.

We also identified a brief questionnaire that owners and veterinarians could use as a rapid screening tool during examinations of geriatric cats (Supplementary Table 5) to act as an early indicator of frailty risk. This screening tool closely maps onto the five domains recognized in phenotypic studies of human frailty, including unintentional weight loss, self-reported exhaustion, low physical activity, weakness, and slow walking speed (34). Further, associations were identified between frailty classifications from this tool and 6-month mortality, suggesting that such an assessment may have clinical significance for senior pet cats and their owners.

The concept of frailty has been extensively studied in human populations. It can predict mortality, disability, hospitalization, length of stay, post-surgery complications, and other outcomes, particularly in high-risk clinical situations such as oncology or surgery (6). Such studies, where patients are assessed in high-risk clinical settings, are arguably not relevant for the current study, where the frailty measure was assessed in a primary care setting. Comparisons with studies assessing frailty in a community health setting are, arguably, more appropriate. The degree of frailty has been assessed in older adults and used to design a treatment plan with two goals: first, to increase the physiological reserve of the patient and build robustness and resilience, and second, to prevent or mitigate stressors (6). Ideally, the aim of care for non-frail older adults is to strive to increase physiological reserve through a healthy lifestyle, management of chronic disease, and preventive care (6). Despite these efforts, the effectiveness of the use of a routine screening tool and subsequent treatment strategy in community medical practice has yet to be fully supported by research outcomes. There have been variable results in studies on routine screening for frailty in humans. In a meta-analysis of 8 studies conducted in the Netherlands, there was no improvement in functional status, quality of life, or clinical outcomes at 1 year (35). However, a study from a community setting in England observed reductions in length of stay, in-hospital mortality, 30-day readmission rate, and institutionalization during the year after the assessment (36). The current study also examined individuals in a community setting, albeit that they were domestic pet cats. The ability to identify frail cats will allow for the development of plans for appropriately treating these cats. One option would be to use our FFQ as a screening test for use by primary care veterinarians, with cats identified being referred to a specialist clinic where a comprehensive geriatric assessment could be conducted to determine tailored intervention targets.

Although research into the frailty in pet cats is limited, there has been some recent research in pet dogs that is of comparative significance, including the development of a frailty index (24) and different “frailty phenotype assessment tools” for senior dogs (21, 23–25). In the most recent of these studies (25), involving dogs enrolled in a longitudinal study of neuro-aging at a university veterinary school, the authors attempted to develop a tool that would be easy to apply to a clinical setting and would also predict all-cause mortality. The tool was developed using a two-phase approach (25). In phase one, a method was created, utilizing retrospective data gathered from 51 dogs to evaluate five frailty domains; in phase two, the tool was evaluated and refined using 198 dogs (25). Overall, the frailty phenotype was positively associated with mortality over a 6-month follow-up period (hazard ratio 4.7).

There are similarities and differences between this dog study and the current study in cats. Both used a combination of owner and veterinarian information and classified frailty across different domains, although these differed between the studies: in the Russell et al. study (25), the final measures were body condition score (BCS), appetite, engagement of the dog with activities, exhaustion, and mobility; in contrast, the current study used assessments of cognitive function, behavior, activity, body weight, BCS, MCS, unexplained weight changes, eating behavior and presence of chronic disease. Therefore, the systems are not directly comparable. Further, although both used a two-stage approach in tool development, there were again differences in the exact approach. In the current study, models of frailty were developed using a training dataset, and performance was tested on a separate test dataset; the previous dog study (26), the tool again used a training dataset, but its ability to predict 6-month mortality was assessed in a second population, after some refinements to improve the tool. The use of 6-month mortality as an outcome measure is a particular strength of the canine study, with the associations identified with their frailty index confirming its clinical importance. Although the current work in cats used owner and veterinary classification of frailty as the primary outcome measure, the veterinarian classification was then tested against 6-month mortality data.

A possible reason for the difference in mortality between the dogs in the Russell et al. (25), study and the cats of the current study, could be that dogs are shorter-lived, on average, than cats. Indeed, in a North American study, the median age at death of pet dogs was 11.6 y (inter-quartile range [IQR] 8.4 y, 13.9 y), compared with a median age in cats of 12.3 y (IQR 7.0 y, 15.7 y) (37). However, differences in lifespan are unlikely to explain the difference in mortality between studies, not least because the dogs in the Russell et al. study (21) were, on average, younger (median 12 years, range 9–17 years) than the cats of the current study (median 14 years, range 10–20). As a result, it is unlikely that the expected remaining lifespan of both populations would have been markedly different.

The associations between frailty classification and mortality suggest that the tool may have clinical merit. However, the mortality data from the two studies suggest that the populations, and therefore findings, might not be directly comparable. In this respect, although frailty was more common (54%) in the current cat study compared with the previous dog study (42%) (25), the 6-month mortality in the cat study was less than half that of the dog study (12% vs. 28% in phase two) (25). This is, perhaps, not unexpected, given the differences in the populations assessed. Whereas the current cat study recruited cats from feline practices, the dog study recruited dogs for cognitive testing in the last 25% or beyond their expected lifespan (25). This population might have been at an advanced stage of aging, explaining this mortality difference. Additionally, dogs were recruited from specialty practices at a single institution, whereas cats were recruited from feline practices across the USA, which may also have influenced results.

The development of an effective screening tool for feline frailty will facilitate the study of cat frailty interventions. Eighty percent of cats identified as frail were alive 6 months later, meaning that the identification of effective interventions could improve the quality of life of large numbers of aging cats.

In humans, physical activity is suggested to prevent or mitigate frailty (38). This is in part due to the negative effects of inactivity on both human physical and mental health. Increased physical activity is associated with “successful aging” in human adults (39). Problem-solving and reasoning tasks are also suggested as an intervention for the cognitive domain of frailty in humans (40).

Therefore, we propose there may be potential benefits for adult and senior cats in maintaining a routine practice of interactive play with wand (“fishing pole”) toys (41), as well as opportunities for problem-solving via food puzzles (42) or activities such as positive reinforcement-based training (43). Further, a lack of interest in play or other enrichment could be an indicator of physical or cognitive challenges that may be associated with frailty (44). Future research could explore the effects of the environment and multimodal environmental modification (45) on assessments of frailty in older cats.

Another area of active investigation to prevent or mitigate frailty is nutrition. In humans, a recent meta-analysis identified the benefits of nutritional management interventions for frail and pre-frail older adults (46). There is less information about nutritional management interventions available for cats, but some recommendations have been made. For example, guidelines promulgated by the American Association of Feline Practitioners and the AAHA address nutrition (28, 47). Additionally, Churchill and Eirmann (48) recently recommended regular, individually-tailored nutritional assessments and recommendations due to the individual age-associated changes in body composition, nutrient needs, and morbidity to ensure the nutrition plan continues to meet the needs of elderly pets at risk for age-related health problems. Unfortunately, no nutrient profile specifically for geriatric cats has been established, so the nutrient composition of products marketed for these cats varies widely. Feeding management is also an essential part of nutritional care and represents part of the owner's bond with the pet. However, shifting the focus from eating the so-called “right food”, to nurturing the cat with foods that provide compassionate care and comfort increases in importance.

Study-related limitations restricted data analysis to baseline questionnaires only. However, the results of the analyses demonstrated the feasibility of the statistical approach to reduce variables and develop models to be tested in subsequent definitive studies. Owner questionnaire variables were reduced from >45 to 14 in the univariate analysis, with three being included in the best-fitting final multivariable model; the variables in the veterinarian questionnaire were reduced from 14 to nine in the univariate analysis, with three being included in the best-fitting final multivariable model (Supplementary Table 5). Of course, further work is now required to determine whether the variables identified best highlight the onset and progression of frailty in cats. Another limitation of the current work was how consistently owners could identify the variables. As with humans, cats can hide their pain and illness or show only subtle signs (49), which can create challenges for owners and clinicians trying to identify frailty (29). Moreover, owners might have difficulty identifying changes that develop too slowly to be readily observed. A further study limitation was the fact that the questions asked to owners were somewhat subjective, with the potential for responses to be affected by how the terms were interpreted as well as owner attitudes and beliefs. This concern was partly addressed by using expert consensus to select variables for inclusion and using readability statistics to ensure that the questionnaire could be understood. Nonetheless, further validation work could be considered, for example, testing the terminology used in owner focus groups to ensure that the meanings are precise, understood by owners, and consistent. A further limitation was the fact that relatively few pedigree cats were included and, therefore, the study findings are, arguably, most applicable to non-pedigree cats. Further work would be required to determine how generalizable the findings are to all pedigree breeds. A final limitation was the fact that the age of the cats was reported by the owner, and no attempt was made to verify this (e.g., by checking veterinary patient records or pedigree documents). Although these data are likely to be accurate for some cats (e.g., those where the date of birth is known), it might have been estimated for other cats, not least those that were rescued or had been rehomed. Further, this metric might have been subject to recall bias, not least given that the median owner-reported age was 14 y. Nonetheless, definitions of aging in cats are not clearly defined; indeed, the authors of the 2021 AAFP Senior Care Guidelines (28) state that “The Task Force feels that ‘geriatric' is more a statement of health status, and has no specifically associated age.” Therefore, although there might have been some inaccuracy in actual cat age, it is likely that owners' opinions on other aspects of the frailty will still be reliable.

One goal of this study was to promote a discussion of similarities and differences in frailty perceptions between owners and clinicians. A recent study of dental pain in cats suggested some useful clues owners and practitioners could use when dental pain was suspected (even if cats hid behavioral signs of pain), which might be adapted to other body systems in geriatric cats (50). These concepts could be further developed to increase multi-modal approaches to better our understanding of geriatric cat welfare and health in the home and clinic environments.

In conclusion, this pilot study demonstrated the feasibility of developing a brief FFQ, providing useful groundwork for a larger definitive trial of its effectiveness in reducing frailty in geriatric cats. In the interim, it can serve as a screening tool in primary care practices to facilitate conversations about geriatric cat care to improve their quality, if not length, of life.

## Data Availability

The original contributions presented in the study are included in the article/Supplementary material, further inquiries can be directed to the corresponding author.

## In memoriam

Dedicated to the memory of Dr. Elizabeth Colleran, a beloved feline veterinarian, colleague, and friend of the authors.

## Supplementary material

Supplemental File 1 Owner questionnaire. [word document].

Supplemental File 2 Veterinarian questionnaire. [word document].

Supplemental File 3 Details of scoring of variables used in the owner and veterinarian prediction models. [word document].

Supplemental File 4 Final dataset used for statistical analyses. [excel file].

Supplemental File 5 Code used for statistical analysis [PDF file].

Supplemental File 6 Sensitivity, specificity, positive predictive value (PPV), and negative predictive value (NPV) based on different frailty thresholds from the training set for the final veterinarian model.
