# Supplementary material for: A non-randomized pilot study to test the feasibility of developing a frailty scale for pet cats
Source: Front Vet Sci. 2025 Feb 26;12:1549566. doi: 10.3389/fvets.2025.1549566 (PMC11897749; doi:10.3389/fvets.2025.1549566)
Supplement: Supplemental File 2 — Veterinarian questionnaire. [word document]. [file Data_Sheet_2.docx]

**Supplemental file 2**

*Final veterinarian questionnaire*

Q1 Client Last Name

Q2 Cat Name

Q3 Cat's weight (please express with not more than 1 decimal)

Q4 Please specify weight units:

- Kilograms/Metric
- Pounds/Imperial

Q5 Has this cat experienced a recent change of >5% of body weight?

- Yes
- No
- Don't know

Q6 If yes, Change in weight:

- Weight loss
- Weight gain

Q7 Cat's BCS (out of 9)

Q8 Has this cat experienced a recent change of >1 point of Body Condition Score (out of 9 point scale)?

- Yes
- No
- Don't know

Q9 If yes, Change in BCS:

BCS Increase

BCS Decrease

Q10 Cat's MCS

- Normal muscle mass
- Mild muscle loss
- Moderate muscle loss
- Severe muscle loss

Q11 Has this cat experienced a recent change of Muscle Condition Score (out of 4 point scale)?

- Yes
- No
- Don't know

Q12 If yes, Change in MCS:

- MCS Increase
- MCS Decrease

Q13 How would you describe this cat's claw condition?

- Normal/healthy
- Overgrown/thickened
- Ingrown
- N/A - cat is declawed

Q14 Does this cat have any of the following illnesses/diseases? *(conditions/problems identified from the history, physical examination and any clinical evaluation deemed appropriate)*

|  | **Yes** | **No/Don't Know** | **Further diagnostics recommended** |
| --- | --- | --- | --- |
| Cancer |  |  |  |
| Neurologic disorder |  |  |  |
| Chronic pain |  |  |  |
| Cognitive dysfunction syndrome |  |  |  |
| Dental disease |  |  |  |
| Dermatologic disease |  |  |  |
| Gastrointestinal disease |  |  |  |
| Heart disease |  |  |  |
| Hyperthyroidism |  |  |  |
| Hypothyroidism |  |  |  |
| Chronic kidney disease |  |  |  |
| Lower urinary tract disorder |  |  |  |
| Endocrine disorders (e.g., diabetes mellitus) |  |  |  |
| Systemic hypertension |  |  |  |

Q15 Please provide detail about any diagnosed illnesses (e.g., kidney disease IRIS stage, type of endocrine disorder, etc).

|  | **Diagnosis/type** | **Date of diagnosis** | **Controlled or cured? Y/N** |
| --- | --- | --- | --- |
| Cancer |  |  |  |
| Neurologic disorder |  |  |  |
| Chronic pain |  |  |  |
| Cognitive dysfunction syndrome |  |  |  |
| Dental disease |  |  |  |
| Dermatologic disease |  |  |  |
| Gastrointestinal disease |  |  |  |
| Heart disease |  |  |  |
| Hyperthyroidism |  |  |  |
| Hypothyroidism |  |  |  |
| Chronic kidney disease (x20) |  |  |  |
| Lower urinary tract disorder |  |  |  |
| Endocrine disorders (e.g., diabetes mellitus) |  |  |  |
| Systemic hypertension |  |  |  |

| **Q16 In the last 3 months, this cat has:** | **Yes** | **No** | **Don't know** |
| --- | --- | --- | --- |
| Involuntarily lost weight |  |  |  |
| Been more fatigued |  |  |  |
| Shown increased cognitive difficulties |  |  |  |
| Shown signs of cognitive dysfunction |  |  |  |

Q17 - Frailty is present in cats showing behavioral changes (like increased meowing, getting lost in the house or aimless wandering, not using the litter box, and restlessness), decreased activity, unexplained changes in weight, and loss of muscle and strength (not climbing or jumping as when younger) that are not explained by a medical problem. The number of health problems also seems to increase in frail cats. The more of these features the cat shows, the more likely they are to be frail. Based upon my assessment, this cat:

- Is not frail (1)
- Is somewhat frail (2)
- Is definitely frail (3)

Q17 Do you have any comments about this cat or the survey?
