# Supplementary material for: A non-randomized pilot study to test the feasibility of developing a frailty scale for pet cats
Source: Front Vet Sci. 2025 Feb 26;12:1549566. doi: 10.3389/fvets.2025.1549566 (PMC11897749; doi:10.3389/fvets.2025.1549566)
Supplement: Supplemental File 3 — Details of scoring of variables used in the owner and veterinarian prediction models. [word document]. [file Data_Sheet_3.docx]

**Supplemental File 3**

*Scoring of variables used in prediction models*

**Owner models**

The following variables were used to develop a multivariable prediction model of frailty using client questionnaire responses:

- Cat age (<16 years, > 16 years)
- Hesitates or avoids climbing or jumping up onto or down from objects
- Grooms less than usual
- Changes in eating habits over the past three months
- Responds slower
- *Behavior composite score 1*. The sum of the following 16 behaviors that clients noticed changes in, adding one to the composite score for each one that was marked as “yes”:
  - plays with toys less
  - moves without purpose
  - stares into space
  - gets lost at home
  - avoids interactions
  - clings to you
  - cries out loudly
  - acts more fearful
  - acts agitated or restless
  - cries when picked up
  - poops outside the litterbox
  - pees outside the litterbox
  - plays less
  - sleeps more
  - explores less
  - active at night.
- *Behavior composite score 2*. The sum of the following 12 behaviors that clients noticed changes in, adding one to the composite score for each one that was marked as “yes”:
  - plays with toys less
  - moves without purpose
  - stares into space
  - gets lost at home
  - cries out loudly
  - acts more fearful
  - cries when picked up
  - poops outside the litterbox
  - pees outside the litterbox
  - plays less
  - sleeps more
  - explores less.

| **Owner questionnaire training set** | | |
| --- | --- | --- |
| **Intrinsic capacity *– Prompt - my cat...*** | | |
| **Abbreviation** | **Qualtrics question** | **Scoring** |
| Cat age | Cat's age in years (round to nearest year) | <16y, >16y |
| Jumps up | Jumps up onto elevated surfaces, like chairs or tables | Empty cell= Never did, Does Not Apply,  Unchanged= Yes, and still does,  Diminished/Ceased= Less than before or not anymore |
| Climbs | Climbs, such as onto furniture or a cat tree |  |
| Uses scratching post | Uses a scratching post |  |
| Good appetite | Has a good appetite |  |
| Sleeps or rests comfortably | Sleeps or rests comfortably |  |
| Looks out window | Looks out the window |  |
| enjoys life | Enjoys life |  |
| **Frailty items – Prompt - Over the past 3 months, have you noticed that your cat:** | | |
| Responds slower | Respond slower than usual when called? | No=No,  Yes=not sure or Yes |
| Hesitates or avoids jumping | Hesitate/avoid jumping up onto or down from objects (for example, onto your lap, chair, or couch)? |  |
| Moves less smoothly | Move less smoothly than usual? |  |
| Grooms less | Groom her/himself less than usual? |  |
| Changes in eating in the previous three months | Eat less food than usual  Eat more food than usual  No significant changes in feeding behavior | No=no Change, Yes=change in eating behavior |
| Changes in weight in the previous three months | Have you noticed any recent changes (gain or loss) in your cat's weight?  No change  Yes, weight gain  Yes, weight loss | No=no Change, Yes=change in weight |
| **Owner frailty assessment** | | |
| Frail? | Do you think your cat is frail? | No=Not Frail, Yes=not sure or Frail |

**Veterinarian models**

For modeling, items from the veterinarian questionnaire included:

- cat age (<16 years, > 16 years)
- muscle condition score
- claw condition
- increase in fatigue or cognitive difficulties in the past three months
- and either a ***chronic kidney disease*** (CKD) binary variable (yes. No)
- or a ***disease composite score***. To construct the disease composite score, responses to fourteen disease binary indicators were assessed, adding one to the composite score for each one that was marked as “yes”; the disease indicators included:
  - cancer
  - neurological disease
  - chronic pain
  - Cognitive Dysfunction Syndrome (CDS)
  - dental disease
  - dermatological disease
  - gastrointestinal disease
  - cardiovascular disease
  - hyperthyroidism
  - hypothyroidism
  - chronic kidney disease
  - chronic lower urinary tract disease
  - endocrine disease
  - hypertension.

| **Veterinarian questionnaire training set** | | |
| --- | --- | --- |
| **Abbreviation** | **Qualtrics question** | **Scoring** |
| Cat age | Cat's age in years (round to nearest year) | <16,y >16y |
| Muscle Condition Score | Cat's Muscle Condition Score (MCS) | Normal=normal muscle mass or mild muscle loss,  Muscle Loss=moderate or severe muscle loss |
| Claw condition | How would you describe this cat's claw condition? | Normal =normal/healthy, Abnormal=overgrown/thickened, ingrown,  N/A = the cat is declawed |
| **Frailty items – Prompt - In the past 3 months, has this cat:** | | |
| Involuntarily lost weight | | Empty cell=Missing, Yes=Yes. No=No/Don't Know. |
| Been more fatigued | |  |
| Shown increased cognitive difficulties | |  |
| Shown signs of cognitive dysfunction | |  |
| **Disease composite score** | | |
| Score derived from 14 binary disease indicated | | Calculated as describe above |
| **Veterinary frailty assessment** | | |
| Based upon my assessment, this cat is: | | Empty cell=Missing, Yes=somewhat/definitely frail, No=not frail |
