# Supplementary material for: A non-randomized pilot study to test the feasibility of developing a frailty scale for pet cats
Source: Front Vet Sci. 2025 Feb 26;12:1549566. doi: 10.3389/fvets.2025.1549566 (PMC11897749; doi:10.3389/fvets.2025.1549566)
Supplement: Supplemental File 5 — Code used for statistical analysis [PDF file]. [file Data_Sheet_5.pdf]

```r
################################################################################
# Script Name: Logistic Regression with Firth Correction and Variable Selection
# Author: Yunyi Ren <yyren@ucdavis.edu>
# Date: 2024/12/17
# Purpose: Perform logistic regression analysis using Firth correction,
#          sensitivity analysis, and variable selection for frailty survey dataset.
# Dependencies: Requires the following R packages:
#   - tidyverse
#   - readxl
#   - MASS
#   - regclass
#   - logistf
#   - pROC



########## Readin Data
data_all_cat <-
readxl::read_xlsx("S:/Research/CTSC/Biostatistics/ActiveCTSC/Yunyi/CTSC_6693_Buffington/Analysis/CTSC_6693_Code/deidentified_all_cats.[illegible]
# Data with both client survey only
data_client <- data_all_cat %>% filter(survey_group == "client")
# Data with both dvm only
data_dvm <- data_all_cat %>% filter(survey_group == "dvm")
# Data with both client and dvm survey
data_both <- data_all_cat %>% filter(survey_group == "both")

########## Generate Test and Training Dataset
# 70% of data with both survey become train
data_both_train <- data_both %>% group_by(combined_frailty) %>% sample_frac(.7)
train_inx = sort(which(data_both$`cat_id`%in%data_both_train$`cat_id`))
test_inx = (1:nrow(data_both))[-train_inx]
# 70% of data with both survey plus client/dvm only survey become train for each survey
Client_train_plus = rbind(data_both_train,Client_Test)[,c(1:47)]
DVM_train_plus = rbind(data_both_train,DVM_Test)[,c(1:2,48:70)]
# 30% of data with both survey become test
data_both_test = na.omit(data_both[test_inx,])


########## Logistic Regression with Firth Correction
model <- glm(formula(), family = "binomial", data = Client_train_plus)
junk <- capture.output(selected_model1 <- MASS::stepAIC(model,direction = "both",k=log(n)))
regclass::VIF(selected_model1)
tf_model1 <- logistf::logistf(data=Client_train_plus, formula())

summary_tf_model1 = cbind(tf_model1$coefficients,sqrt(diag(tf_model1$var)),tf_model1$prob)
colnames(summary_tf_model1)  = c("Estimate","Std. Error","Pr(>|z|)")
oddtb <- as.data.frame(summary_tf_model1)
oddtb$`Odd Ratio` = round(exp(oddtb$Estimate),2)
oddtb$`Upper 95% CL` = round(exp(oddtb$Estimate + qnorm(0.975)* oddtb$`Std. Error`),2)
oddtb$`Lower 95% CL` = round(exp(oddtb$Estimate - qnorm(0.975)* oddtb$`Std. Error`),2)
oddtb$`Pr(>|z|)` = round(oddtb$`Pr(>|z|)`,4)
oddtb$`Estimate` = round(oddtb$Estimate,3)
oddtb[1,] = c(round(oddtb$Estimate[1],3),rep("",dim(oddtb)[2]-1))
oddratiotb1 <- oddtb[,c(1,4,6,5,3)]

data_both_test$prob_client1 = predict(tf_model1,newdata = data_both_test,type="response")
Client_train_plus$prob_client1 = predict(tf_model1,newdata = Client_train_plus,type="response")

# Sensitivity Analysis
roc_obj1 <- pROC::roc(Client_train_plus$Frail, Client_train_plus$prob_client1)
train_roc_obj1 <- pROC::roc(Client_train_plus_$Frail, Client_train_plus_$prob_client1)
coords_with_tpv_fpv <- pROC::coords(train_roc_obj1, "all", ret=c("threshold","sensitivity","specificity","ppv","npv"))

########## Functions used for variable selection
# 1. CohenKappa()
# 2. Chisq_pvalue()
# 3. PointBiserial()
# Compute Cohen's Kappa and confidence intervals for all pairwise comparisons of columns in a dataframe.
CohenKappa <- function(df, alpha = 0.05) {
  result <- data.frame(Var1 = character(),
                       Var2 = character(),
                       Estimate = numeric(),
                       SE = numeric(),
                       LL = numeric(),
                       UL = numeric(),
                       P.Value = numeric(),
                       Tukey.P.Value = numeric(),
                       stringsAsFactors = FALSE)

  for (i in 1:(ncol(df) - 1)) {  # Loop through pairwise column combinations
    for (j in (i + 1):ncol(df)) {
      temp <- as.numeric(table(df[, c(i, j)]))
      kappa_ci <- statpsych::ci.kappa(alpha,
                                      f00 = temp[1],
                                      f01 = temp[2],
                                      f10 = temp[3],
                                      f11 = temp[4])[2, ]
      result <- rbind(result, data.frame(
        Var1 = names(df)[i],
        Var2 = names(df)[j],
        Estimate = round(kappa_ci[1], 3),
        SE = round(kappa_ci[2], 3),
        LL = round(kappa_ci[3], 3),
        UL = round(kappa_ci[4], 3),
        P.Value = NA,
        Tukey.P.Value = NA
      ))
    }
  }

  result <- result %>%  # Add p-values and Tukey-adjusted p-values
    mutate(
      P.Value = round(pt(Estimate / SE, df = nrow(df), lower.tail = FALSE), 4),
      Tukey.P.Value = round(ptukey(Estimate / SE, nmeans = nrow(result), df = nrow(df), lower.tail = FALSE), 4)
```

```r
    ) %>%
    arrange(Tukey.P.Value, P.Value)

  return(result)
}

# Compute Chisqure's P-value for all variables in data df, return the p-value matrix.
Chisq_pvalue <- function(df, cat.index) {
  result_table <- data.frame(Var1 = character(),
                             Var2 = character(),
                             Chisquared_P_Value = numeric(),
                             stringsAsFactors = FALSE)

  bi.index <- setdiff(1:ncol(df), cat.index)
  for (i in cat.index) {
    for (j in bi.index) {
      if (length(unique(df[, j])) > 1 & length(unique(df[, i])) > 1) {
        p_value <- chisq.test(df[, i], df[, j])$p.value
        result_table <- rbind(result_table,
                              data.frame(Var1 = names(df)[i],
                                         Var2 = names(df)[j],
                                         Chisquared_P_Value = round(p_value, 4)))
      }
    }
  }

  return(result_table)
}

# Calculates point-biserial correlation between continuous and categorical variables,
# and Pearson correlation between continuous variables in a dataframe
PointBiserial <- function(df,cont.index){
  tb <- matrix(0,ncol = 3)
  tb <- as.data.frame(tb[-1,])
  cat.index = (1:ncol(df))[-cont.index]

  if (length(cont.index)>1){
    for (i in 1:(length(cont.index)-1)){
      for (j in (i+1):length(cont.index)){
        corr <- cor(df[,i],df[,j])
        tb <- rbind(tb,
                    c(names(df)[i],
                      names(df)[j],
                      round(corr,3))
        )
      }
    }
  }

  for (i in cont.index){
    for (j in cat.index){
      df_ = na.omit(df[,c(i,j)])
      corr <- ltm::biserial.cor(df_[,1],df_[,j2])
      tb <- rbind(tb,
                  c(names(df_)[1],
                    names(df_)[2],
                    round(corr,3))
      )
    }
  }
  names(tb) <- c("Var1","Var2","Point-Biserial Correlation")
  return(tb)
}
```
