# Supplementary material for: A non-randomized pilot study to test the feasibility of developing a frailty scale for pet cats
Source: Front Vet Sci. 2025 Feb 26;12:1549566. doi: 10.3389/fvets.2025.1549566 (PMC11897749; doi:10.3389/fvets.2025.1549566)
Supplement: Supplemental File 6 — Sensitivity, specificity, positive predictive value (PPV), and negative predictive value (NPV) based on different frailty thresholds from the training set for the final veterinarian model. [file Data_Sheet_6.docx]

**Supplemental File 6**

**Sensitivity, specificity, positive predictive value (PPV), and negative predictive value (NPV) based on different frailty thresholds from the training set for the final veterinarian model.**

| **Frailty threshold** | **Sensitivity** | **Specificity** | **PPV** | **NPV** |
| --- | --- | --- | --- | --- |
| 0.000 | 1.000 | 0.000 | 0.404 | - |
| 0.102 | 1.000 | 0.115 | 0.433 | 1.000 |
| 0.152 | 0.954 | 0.333 | 0.492 | 0.914 |
| 0.220 | 0.877 | 0.573 | 0.582 | 0.873 |
| 0.308 | 0.723 | 0.750 | 0.662 | 0.800 |
| 0.412 | 0.646 | 0.938 | 0.875 | 0.796 |
| 0.524 | 0.585 | 0.979 | 0.950 | 0.777 |
| 0.665 | 0.569 | 0.979 | 0.949 | 0.770 |
| 0.787 | 0.523 | 0.979 | 0.944 | 0.752 |
| 0.854 | 0.462 | 0.990 | 0.968 | 0.731 |
| 0.902 | 0.431 | 1.000 | 1.000 | 0.722 |
| Results are for multiple regression analyses using the final veterinarian model (including the disease composite score), where different frailty thresholds derived from the veterinarian training set were applied to the veterinarian validation set. | | | | |

**Final Client Questionnaire**

*Q1.* This is a study to help us learn more about how cats age. You have been invited to participate because you currently have a cat between 11 and 20 years of age. You must be 18 years of age or older to participate. There are no risks or benefits to you in participating in this survey. You may choose to participate or not. You may answer only the questions you feel comfortable answering, and you may stop at any time. Completion of the survey indicates your consent to the above conditions.

The survey should take approximately 15 minutes to complete. Any questions or concerns should be directed to the study veterinarian: Dr. Tony Buffington, DVM at [*drbuffington@ucdavis.edu*](mailto:drbuffington@ucdavis.edu)*.* PLEASE NOTE THAT YOUR VETERINARIAN WILL REVIEW YOUR ANSWERS and may discuss them with you. Thank you for participating in this study about your cat. When you have completed the survey, please be sure to enter your email so we can send you a gift card as compensation!

*Q2.* Your Last Name

*Q3.* Your cat's name

*Q4.* Your cat's Breed

- Domestic Shorthair
- Domestic Medium/Longhair
- Other:

*Q5.* Your cat's age in years (round to nearest year)

*Q6.* How long have you owned your cat (closest number in years)?

*Q7.* What is your cat's sex?

- Male
- Female

*Q8.* Is s/he spayed or neutered?

- Yes
- No

*Q9.* Please respond to these statements about your cat. Choose the answer that best describes whether your cat engages in the described activity.

| **My cat...** | **Never Did** | **Yes, and still does** | **Yes, but less than before** | **Not any more** | **Does not apply** |
| --- | --- | --- | --- | --- | --- |
| Plays with toys |  |  |  |  |  |
| Jumps up onto elevated surfaces, like chairs or tables |  |  |  |  |  |
| Climbs, such as onto furniture or a cat tree |  |  |  |  |  |
| Uses a scratching post |  |  |  |  |  |
| Has a good appetite |  |  |  |  |  |
| Comes to me for touching/petting |  |  |  |  |  |
| Cuddles with me/sits on my lap |  |  |  |  |  |
| Sleeps or rests comfortably |  |  |  |  |  |
| Sleeps or rests in warm spots in the home |  |  |  |  |  |
| Shares the same room with his/her people to rest or interact |  |  |  |  |  |
| Goes outdoors |  |  |  |  |  |
| Looks out the window |  |  |  |  |  |
| Enjoys catnip or other treats |  |  |  |  |  |
| Enjoys life |  |  |  |  |  |

| ***Q10.* Over the past 3 months, have you noticed your cat:** | **Yes** | **No** | **Don't Know** |
| --- | --- | --- | --- |
| Move (pace, walk in circles, or wander) without purpose? |  |  |  |
| Stare into space? |  |  |  |
| Get lost in your home? |  |  |  |
| Walk away from interactions with people or other animals? |  |  |  |
| Avoid interactions with you or others? |  |  |  |
| Cling to you more than usual? |  |  |  |
| Cry out loudly for no apparent reason? |  |  |  |
| Act more fearful than usual? |  |  |  |
| Act more agitated or restless than usual? |  |  |  |
| Startle more easily than usual? |  |  |  |
| Respond slower than usual when called? |  |  |  |
| Hesitate/avoid jumping up onto or down from objects (for example, onto your lap, chair or couch)? |  |  |  |
| Limp? |  |  |  |
| Move less smoothly than usual? |  |  |  |
| Use stairs slowly/one at a time? |  |  |  |
| Cry when picked up or petted? |  |  |  |
| Defecate (poop) outside the litterbox? |  |  |  |
| Urinate (pee) outside of the litterbox? |  |  |  |
| Groom her/himself less than usual? |  |  |  |
| Play less than usual? |  |  |  |
| Sleep more than usual? |  |  |  |
| Act less interested in exploring your home than usual? |  |  |  |
| Act more active at night than usual? |  |  |  |

| ***Q11.* How frequently do you observe these behaviors?** | **Rarely** | **Sometimes** | **Often** |
| --- | --- | --- | --- |
| Walk away from interactions with people or other animals? |  |  |  |
| Respond slower than usual when called? |  |  |  |
| Hesitate/avoid jumping up onto or down from objects (for example, onto your lap, chair or couch)? |  |  |  |
| Limp? |  |  |  |
| Move less smoothly than usual? |  |  |  |
| Use stairs slowly/one at a time? |  |  |  |

*Q12.* Over the past 3 months, have you noticed your cat:

- Eat less food than usual
- Eat more food than usual
- Still eating the same amount as usual

*Q13.* Have you noticed any recent changes (gain or loss) in your cat's weight?

- No change
- Yes, weight gain
- Yes, weight loss

*Q14.* Aging cats may show behavioral changes (like increased meowing, getting lost in the house or aimless wandering, not using the litter box, restlessness, or decreased activity), unexplained changes in weight, and loss of muscle and strength (not climbing or jumping as when younger) that are not explained by a medical problem. The more of these features the cat shows, the more likely they are to be frail. The number of health problems also seems to increase in frail cats.

Do you think your cat is frail?

- Yes
- No
- Not sure

*Q15.* Please enter your email address so we can send you your gift card when you complete the study as a thank you for your participation. Your email address will be stored securely and only used to send you an electronic gift card. If you do not wish to enter your email address, you can skip this question.

17 Do you have any comments about this cat or the survey?
